# Electromyography Assessment of the Assistance Provided by an Upper-Limb Exoskeleton in Maintenance Tasks

**DOI:** 10.3390/s19153391

**Published:** 2019-08-02

**Authors:** Andrea Blanco, José María Catalán, Jorge Antonio Díez, José Vicente García, Emilio Lobato, Nicolás García-Aracil

**Affiliations:** 1 Department of Systems Engineering and Automatic, Miguel Hernández University, 03202 Elche, Spain; 2 MovilFrio S.L., 03006 Alicante, Spain

**Keywords:** exoskeleton, upper limb, design, musculoskeletal injuries, validation, AnyBody™, electromyography

## Abstract

In this paper, the analysis of the intensity of muscle activations in different subjects when they perform an industrial task in a repetitive way assisted by a robotic upper-limb exoskeleton is presented. To do that, surface electromyography (EMG) signals were monitored with and without a robotic upper-limb exoskeleton for 10 subjects during a drilling task, a typical tedious maintenance or industrial task. Our results show that wearing the upper-limb exoskeleton substantially reduces muscle activity during a drilling task above head height. Specifically, there is statistically significant differences in the pectoralis major and rhomboids muscles between the groups wearing or not wearing the robotic upper-limb exoskeleton.

## 1. Introduction

In the Sixth European Working Conditions Survey [1] major physical risks at work were studied and analyzed, among which are ergonomic risks. These are the most prevalent risks in Europe—in particular, repetitive hand and arm movements (62% of workers report this)—and include the musculoskeletal disorders that can play a role in common workplace complaints, affecting millions of workers and costing billions of euros to employers.

Musculoskeletal injuries are a collection of painful disorders of muscles, ligaments, tendons, etc., which affect, above all, the neck, upper limbs, and back, although they can affect all body parts [2].

Exoskeletons are a well-accepted solution to carry out repetitive hand and arm movements in several industrial domains, reducing loads on operators in critical workflows and thus avoiding musculoskeletal disorders without hampering the workers [3], although their large-scale implementation in industry has still a long way to go.

A robotic exoskeleton system is an orthotic and wearable mechanical structure with corresponding joints and links to the human ones. It transmits torques to the human joints offering support to the user and therefore improving their motor capacity [4].

So many exoskeletons exist today that is difficult to categorized them. It is possible to consider two main aspects: mechanical structure and control system [5]. From the mechanical point of view, the characteristic of the exoskeleton has been reviewed many times, as in [6,7], where a review of the most recent exoskeleton developments is found. Even so, it is usual to classify them according to the final application such as haptic interaction [8,9], human power augmentations exoskeletons (such as the one proposed in this paper) [10,11], and medical/rehabilitation purposes. The latter has been the main application area of the exoskeletons, where the devices are intended to help people with reduced mobility, such as CADEN-7 [12], WOTAS [13], SUEFUL-7 [14], ARMin III [15], IntelliArm [16], 6-REXOS [17], ETS-MARSE [18], among others. Nevertheless, there is an increasingly significant interest in the development of wearable robots or exoskeletons applicable specifically for industrial purposes.

### 1.1. Exoskeletons in Industry

These mechanisms, as already pointed out, reduce the mechanical efforts that fall on workers when they perform manual tasks, increasing therefore the operator’s capacity and reducing the risk of suffering injuries such as musculoskeletal disorders. In [19,20] you can find a review of exoskeletons designed for industrial purposes, where their effect on reducing the physical load in the human body is evaluated. The authors state that the reviewed papers reported up to 80% of muscle activity reductions as a direct effect of performing hard and repetitive work tasks assisted by active exoskeletons, as opposed to the use of passive exoskeletons that result in a reduction of between 10% and 40%.

Similar devices to the one we proposed in this paper have been found in the literature review, but they are larger and heavier that the one that is intended to be manufactured. Some, such as the FORTIS Exoskeleton by Lockheed Martin [21], have a passive exoskeleton structure for the lower limbs. It does not manipulate the tool with an arm exoskeleton, but does so by means of an external structure. We propose an arm exoskeleton that aims to replicate the natural movement of the human arm and, therefore, makes its use more intuitive than the use of other robotic systems such as those found in the literature reviewed. Others, such as WSAD (Wearable Stopping-Assist Device) [22], consist of a passive structure that reduces possible back injuries, but could not support the weight of the arm exoskeleton.

Although there is great interest in the development of exoskeletons for industrial applications, the MovilFrio company, promoter of the ExIF project, has not found in the market any system that meets the requirements to meet the needs of its employees, and at the same time has seen that there is a real market niche for the systems that are intended to be developed within the framework of this project. This Spanish company is dedicated to performing technical assistance, maintenance, and installation of all types of systems both in construction and industry, among which are air-conditioning systems, automation, or logistics, and is committed to improving the well-being of its workers, promoting adapted schedules that favor reconciliation of professional and family life, and developing a comfortable and safe working environment.

Because of this, the ExIF project arises (Intelligent Robotic Exoskeleton and Advanced Interface Systems Man Machine for Maintenance Tasks in the Industries of the Future), which proposes the development of a robotic upper-limb exoskeleton that will be supported by a passive lower-limb exoskeleton that transmits the supported loads to the ground as they are introduced in [23]. In this way, it is intended to increase the user’s own strength, allowing loads to be handled easily and efficiently, avoiding repetitive movements and the adoption of fatiguing or painful postures during the performance of industrial maintenance work, reducing or eliminating the musculoskeletal disorders described above.

The paper presented below specifically aims to validate a first prototype of the exoskeleton designed for the upper limb by comparing the muscle activity analyzed in a laboratory experiment, as well as to provide more details about the design process and validation in simulation environment of the design that was previously introduced in [23].

### 1.2. Electromyography (EMG)

Electromyography (EMG) is a diagnostic procedure used to evaluate the health of the muscles and nerve cells that control them. There are several ways to capture EMG signals, both invasive and non-invasive, the latter being interesting because of its flexibility, low cost, and less impact on the user compared to invasive sensors.

The use of the EMG signal to describe muscle activity during a specific task is widely employed in the bibliography. The information provided by this signal is employed for multiple purposes [24,25,26,27]. Among the applications most commonly studied within the near fields of study of this article is the design of cognitive Human–Robot Interfaces (cHRIs) to design an EMG-based control for power-assisted exoskeletons for assistive [28,29,30,31] and rehabilitation purposes [32,33], and the study on the effects of muscle activity during a certain activity [34,35].

In this study, EMG signals have been used to estimate the reduction of the muscle load by observing the differences in muscle activity when the exoskeleton exposed in the present work is used to perform a certain activity with respect to not using the exoskeleton. Among the different non-invasive sensors for measuring EMG signals, the Shimmer3 EMG unit [36] has been chosen because it is a widely used and verified device, as can be seen in the previous references.

## 2. Design of the Robotic System

First, it is necessary to establish the requirements that the system must meet. Some of them are typical of this type of device, and others are requirements imposed by MovilFrio, the company that develops the robotic device together with the Miguel Hernandez University of Elche.

Then, a kinematic and dynamic analysis of the human arm is carried out while the user performs the tasks of his/her job, in order to base the design of the exoskeleton on the tasks it is intended to support. For these simulations the program AnyBody™ will be used, which is software that performs a simulation of the mechanics of the body. This program is useful for studying how the human body will behave in different situations, which allows us to know the efforts carried out by the joints when performing various tasks. AnyBody™ has been used for the simulation due to the large number of examples that have been found in which this program is employed, such as [37,38,39,40], and that therefore demonstrates its wide degree of application in this field.

Finally, a first concept of the system is designed and validated in simulation environment with the objective of confirming that the solution, we propose, offers a benefit for the users, reducing the effort supported by them during the execution of industrial tasks.

### 2.1. Requirements

On one hand, there are certain common requirements to exoskeleton-type mechanisms used in industrial applications, among which are those mentioned below:

- The device should be comfortable and it should be as small and light as possible
- Since the mechanism is always in contact with the user, it must be safe for the operator
- The device must be able to perform the natural movements of the human arm, without limiting the user’s range of motion

On the other hand, MovilFrio has a clear view of the final objective of the exoskeleton to be developed: the system developed should allow the average person to work with heavy tools, not noticing the load. To meet this objective, the company has determined the following requirements or technical specifications:

- The device must have a modular architecture, i.e., that the joints are independent of each other, to act actively or passively depending on the needs of the user
- The robotic system should be easy to manipulate for the operator
- The exoskeleton must be able to adapt to different arm configurations (a single system for several people)
- The actuators must offer enough torque to support the weight of the user’s arm together with the work tool (the necessary torque is calculated in later sections of this paper)
- The maximum weight of the device must not exceed 20 kg
- The maximum permissible load in the vertical working position (90° shoulder and elbow flexion) must be 6 kg
- The maximum permissible weight in the load-carrying position with one arm (10° shoulder abduction) must be 8 kg

**Figure 1:**
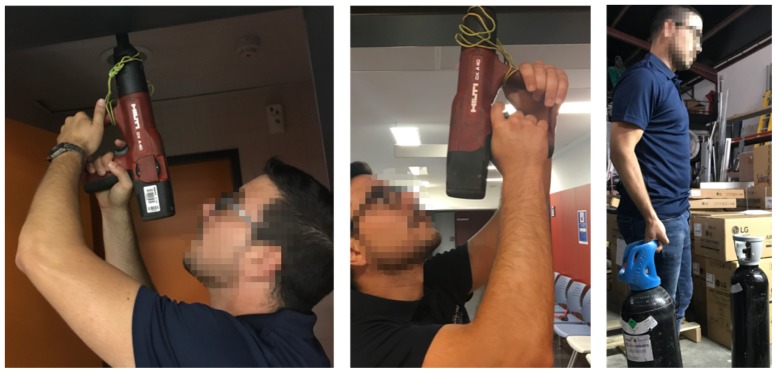
Examples of industrial maintenance tasks: work tasks with the hands overhead in drilling holes into the ceiling and lifting and carrying loads. *All the participants that appear in the figure have provided written and informed consent for its publication.*

Figure 1 shows some of the activities to be carried out by the workers of MovilFrio that can cause musculoskeletal disorders and therefore it is intended to provide support through the manufacture of the exoskeleton.

### 2.2. Kinematic and Biomechanical Analysis of the Human Arm

To model the concept design, we must first know the movements of the upper limb and the maximum range of movement of each of the joints that make up the human arm. Specifically, the proposed device will have 5 DoF to support the following user movements: shoulder abduction/adduction; shoulder flexion/extension; shoulder internal/external rotation; elbow flexion/extension; and wrist pronation/supination.

**Figure 2:**
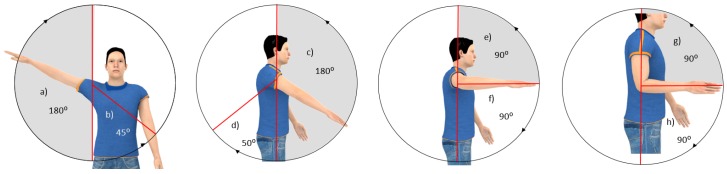
Maximum ranges of movements in the upper limb: (**a**) abduction, (**b**) adduction, (**c**) shoulder flexion, (**d**) shoulder extension, (**e**) external rotation, (**f**) internal rotation, (**g**) elbow flexion, (**h**) elbow extension.

The maximum ranges of each of these movements have been defined based on the information collected in [41], shown in Figure 2. By limiting the design of the mechanism to these maximum ranges, it will be placed on the safety side, since the operator will not exceed these joint limits when performing the tasks of his work.

Taking into account these movements, a first concept design is made where the mechanism can follow the trajectories carried out by the operator through passive joints.

For the analysis of the efforts supported by each of the joints, the recording of some user’s arm trajectories during the accomplishment of target tasks in his/her work environment in which the device is intended to be incorporated has been carried out, such as lifting loads or handling assembly tools.

**Figure 3:**
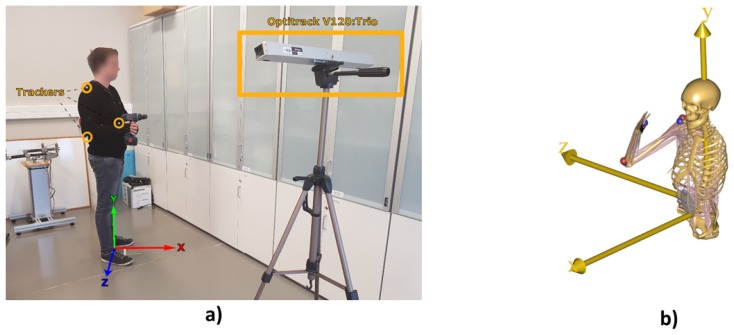
Recording of trajectories simulating one of the tasks to be performed by the operator, where (**a**) shows the setup for recording the trajectories and (**b**) the AnyBody™ model replicating the movements. *The participant that appears in the figure has provided written and informed consent for its publication*.

The recording of these trajectories has been made by using the V120:Trio camera from OptiTrack^®^, which is a tracking camera system with very high-precision infrared technology. These cameras allow the tracking in three dimensions of fixed trackers formed by spherical infrared reflectors. To record the movement made by the upper limb, it has been used a suit specially designed to attach this type of trackers recognizable by the cameras system, as can be seen in Figure 3a, to which has been placed trackers based on the points of kinematic reconstruction that there is defined in AnyBody™, shown in Figure 3b.

Recorded trajectories allow us to define which is the natural movement that describes the arm carrying a certain weight at a specific speed. In this case, the analyzed task consists of lifting a 3 kg drill over the shoulder, where the right arm (arm on which the exoskeleton would be anchored) supports 2 kg, and the left arm would act as a guide and support for the previous one. The duration of the task is approximately 2 s.

Then the recorded trajectories are simulated in AnyBody™, indicating the magnitude of the force and its direction that reflects that the operator is handling a tool. This information can be used to calculate the efforts that are necessary in the joints of the mechanical device to carry out the recorded trajectories, thus being able to estimate which is the torque that each actuator must perform in order to compensate the load that the worker should bear without the help of the robotic system.

On one hand, we have the data obtained in the previous simulation referring to the maximum torques that are needed in each joint to carry out the proposed activity, and on the other hand, we have the data referring to the maximum power generated at each joint.

These values correspond to the effort of the operator when performing the task without the robotic system. Since the simulation was carried out in a phase prior to the design of the mechanism to select the final components and materials of the system, the data obtained does not take into account the real weight of the mechanism. Therefore, a safety factor has been established by which the values obtained in the simulation have tripled, as shown in Table 1, so that in no case the efforts supported by the user related to the weight of the exoskeleton exceed the established values.

### 2.3. Upper-Limb Exoskeleton

Starting from the concept of passive exoskeleton, the design of the active upper-limb exoskeleton that appears in [23] was made, and is detailed below. For this, the actuators were selected based on the results of the previous simulation, and the kinematic analysis of the proposed mechanism was analyzed, which has been useful for its control in later phases.

#### 2.3.1. Kinematic Analysis of the Upper-Limb Exoskeleton

**Figure 4:**
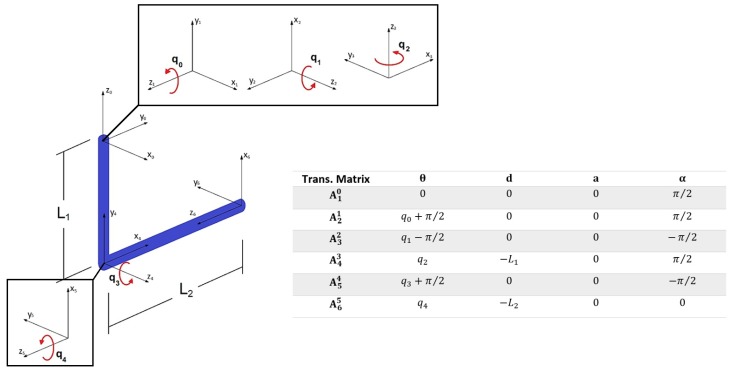
Reference systems and DH parameters of the proposed configuration.

The kinematics of the 5 DoF upper-limb exoskeleton is analyzed using the DH (Denavit–Hartenberg) representation [42]. The reference systems of the mechanism, as well as its DH parameters, are shown in the Figure 4, where $L_{1}$ and $L_{2}$ are the lengths of the upper and the lower arms respectively, which may vary depending on the configuration of the user’s arm. Since the arrangement of the motors in the upper-limb exoskeleton coincide with the user’s arm articulations, the kinematics of the device coincides with the kinematics of the human arm.

On the other hand, the inverse kinematics problem is to find the joint variables given the desired positions and orientations of the end-effector through the inverse mapping. When robot’s morphology imposes constraints about maximum reachable motor angles, such as an exoskeleton robotic device, the inverse kinematics problem is not trivial. Specifically, those constraints are not fixed but variables, and they depend on the arm position of the user in the case of upper-limb exoskeletons. Therefore, a method of calculating the inverse kinematics that changes these limits in a simple way has been developed based on the approach presented in [43]:

(1) $$\begin{array}{c} q^{*}=arg\;min_{q\in R^{n}}\left(∥\alpha_{d}-K_{\alpha }{\left(q\right)∥}^{2}+\beta \cdot {\left(q_{rest}-q\right)}^{T}W\left(q_{rest}-q\right)\right)\\ s.t.\left\{\begin{array}{c} ∥x_{d}-K_{x}{\left(q\right)∥}^{2}<\varepsilon \\ q_{L}<q<q_{U} \end{array}\right. \end{array}$$

where:

- $q^{*}\in R_{5}$: joint coordinates vector that reach a given position
- $x_{d}\in R_{3}$: cartesian coordinates of the end-effector
- $\alpha_{d}\in R_{4}$: orientation quaternion of the end-effector
- $K_{x}\left(q\right)$: forward kinematic function that computes the position of the end-effector from the joint angles *q*
- $K_{\alpha }\left(q\right)$: forward kinematic function that computes the orientation of the end-effector from the joint angles *q*
- $q_{rest}\in R_{5}$: preferred joint configuration
- $W\epsilon R_{5x5}$: diagonal matrix of weighting factors
- β: positive scalar weighting the influence of $q_{rest}$
- ε: maximum permissible error in the position of the end-effector
- $q_{L},q_{U}\in R_{5}$: minimum and maximum permissible joint coordinate vectors

Then, the computation of the inverse kinematics has been converted into an optimization problem, consisting of finding the $q^{*}$ vector that minimizes the above expression, satisfying the constraints. The Matlab function ***fmincon*** is used to solve this problem.

The angle limits have been chosen following the arm’s anatomical limits calculated in [44], and are presented in Table 2:

#### 2.3.2. Selection of Actuators

Since the exoskeleton is designed to assist the operator in installation and maintenance tasks of industrial facilities, the device must support part of the load supported by the operator in real industrial maintenance scenarios. That is why it will be necessary for the mechanism to have certain motor-reducer-type drives that meet specific features and requirements.

These requirements are extracted from the data resulting from previous experiments with the AnyBody™, which are collected in Table 1.

Taking into account the data collected in the previous table, we proceed to select those actuators (motor and gear set) that meet these characteristics, trying to reduce their weight and size as much as possible, to avoid possible collisions and facilitate the manipulation of the mechanism. Electrical motors are the commonly used actuators for the upper-limb exoskeleton robots due to their advantages such as high dynamic, precision and higher controllability using advanced motion control system [6].

In this case, we have selected different sets consisting of actuators from Maxon Motor^®^, attached with strain wave gearing (also known as harmonic gearing). For shoulder movements, both abduction/adduction and flexion/extension, EC90 flat motor have been chosen with a gear with a ratio reduction of 100:1, obtaining an output nominal torque of 44.4 Nm. For the internal/external shoulder rotation, the selected set is an EC45 flat motor with a gear with a ratio reduction of 100:1 smaller than the shoulder set one, but we have incorporated a set of pulleys which add a ratio reduction of 3.5:1, obtaining an output nominal torque of 28.84 Nm. Finally, for the elbow flexion/extension, it has been chosen to use the same actuator as for internal/external rotation, with a reduction ratio of 160:1, so that the output nominal torque is 20.48 Nm.

#### 2.3.3. Adaptability of the Mechanism

As it has been introduced before, it is interesting to consider the possibility of adapting the same device to different arm configuration, so that it can be used only one exoskeleton for different operators. This will not only reduce costs to the company, but will favor the comfort of the user, one of the main drawbacks that currently exist when using a robotic device of the exoskeleton type. To this end, the links have been provided with grooves in order to modify their length, and two commercial linear slides of the HepcoMotion^®^ brand have been incorporated to adapt the shoulder height and thus align the axis of rotation of the exoskeleton with the user’s axis of rotation. In addition, each of these guides is provided with a brake to make the handling and transport of the device comfortable.

The anthropometric dimensions that have been taken as reference for the design of the mechanism are included in the article by [45].

#### 2.3.4. Device Design

**Figure 5:**
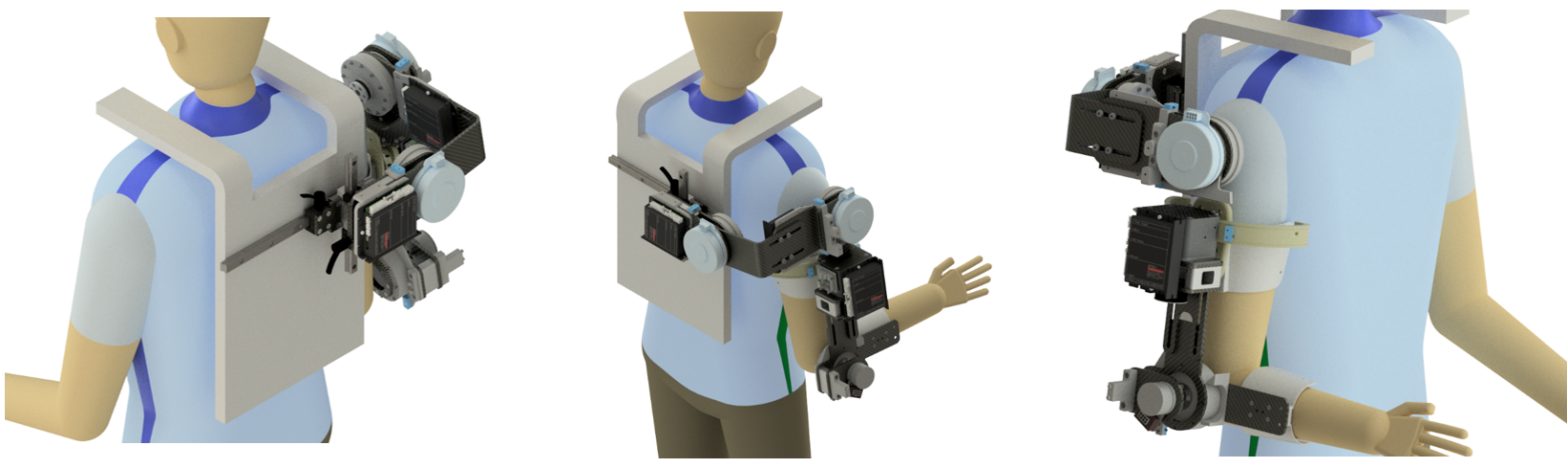
Upper-limb exoskeleton design.

Taking into account all the described requirements, the upper-limb exoskeleton shown in Figure 5 has been designed using Inventor^®^ (software from Autodesk^®^). This program allows analysis of the behavior of the different elements that make up the system based on their material, simulating the deformations that may suffer when applying different loads in the most conflicting points of the structure.

The material selected for the links of the exoskeleton is the carbon fiber, due to the high strength/weight ratio that it provides, with the exception of the link that joins the motors located in the shoulder that will be steel, after analyzing the deformation suffered by the piece as a result of the torsion that appears in it.

On the other hand, the set of pulleys that give rise to the movement of shoulder internal/external rotation will be manufactured in PET-G (polyethylene terephthalate glycol-modified) by 3D printer. Models with versions of the technologies have been made to analyze the resistance to abrasion that the pieces bear due to the contact with the cable that transmits the movement, as well as to verify that the movement made is the desired one.

The connections between the robotic system and the user have been designed based on the shape of the commercial orthosis used by orthopedics, so that the user is as comfortable as possible. They will be manufactured in PLA (polylactic acid) by 3D printing and will be covered inside with EVA foam (ethylene-vinyl acetate), a hypoallergenic material that will be in contact with the skin of the user.

Using these materials an upper-limb exoskeleton with a weight of 9.5 kg is achieved, which makes it lighter than other commercial devices. The weight of the mechanism is theoretical, since it has not been manufactured yet, but it is a good approximation, since all the elements are taken into account. In addition, we must add the weight of the lower-limb exoskeleton and check that the requirement imposed by the company that determines that the robotic system should not exceed 20 kg is not surpassed.

### 2.4. Biomechanical Validation of the Robotic System in Simulation Environment

In this section, we proceed to describe the experimentation carried out through the AnyBody™ program, by means of which we intend to validate the device proposed, demonstrating the benefits of using an upper-limb exoskeleton when it is performed the tasks that have been described throughout this paper. In addition, a passive lower-limb exoskeleton has been incorporated to the simulation to compare whether there really is a reduction in muscle activity when it is assembled to the designed arm exoskeleton. Since this device is not yet developed, the design of a previous concept that will serve as a basis for future versions was used.

For this purpose, the transmitted torques to the corresponding arm joints of the operator are going to be statically analyzed, not only with the exoskeleton but also without the robotic system, comparing the results obtained to determine whether the proposed solution represents an improvement for the company and its workers. To that end, the following load hypothesis have been simulated:

1. Operator loading a tool of 3.1 kg over the shoulder.
2. Operator loading a tool of 3.1 kg over the shoulder with a non-assisted arm exoskeleton.
3. Operator loading a tool of 3.1 kg over the shoulder with an assisted arm exoskeleton.
4. Operator loading a tool of 3.1 kg over the shoulder with an assisted full exoskeleton (upper-limb exoskeleton attached to a lower-limb exoskeleton).
5. Operator loading a tool of 3.1 kg over the shoulder with a full exoskeleton when the arm exoskeleton is blocked.

The model used in the simulation is composed by Shoulder-Arm Model [46], Lumbar-Spine Model [47] and Twente Lower Extremity Model [48]. The boundary conditions in relation to Human–Ground interface are as follows:

- Center of mass of the human body must be kept above the origin of the global reference frame
- Toe and heel must be on the ground for both feet
- Rotation axis of both ankles are aligned
- Both feet are symmetrically placed with respect the body’s sagittal plane

For these restrictions only *b* constraint generates external reaction forces, the rest must be accomplished with body internal forces (muscles) and reactions (joints).

As for the boundary conditions in relation to the Human–Object interface, it should be noted that the object is joined to the hands by means of a revolute joint whose rotation axis is aligned with the extended thumb. On the other hand, regarding the boundary conditions in relation to the Human–Exoskeleton interface, we have the following:

- Forearm interface → cylindrical joint aligned to the forearm’s longitudinal axis, so a non-rigid attachment can be modelled
- Upper-arm interface → cylindrical joint aligned to the upper arm’s longitudinal axis
- When the leg exoskeleton is not implemented → exoskeleton’s back part is attached rigidly to the trunk
- When the leg exoskeleton is implemented → exoskeleton’s back part is attached rigidly to the ground reference system

Since AnyBody™ works with inverse dynamics, to calculate the stresses in the joints is necessary to define the positions of each of them. In this study, they have been specified as below:

- Shoulder abduction/adduction (sA/A) →−0.4009 rad
- Shoulder flexion/extension (sF/E) →−0.37419 rad
- Shoulder internal/external rotation (sI/E) →−0.09553 rad
- Elbow flexion/extension (eF/E) →−2.1746 rad

The results obtained for the simulations performed have been collected in Table 3. This analysis also calculates the maximum percentage of muscle activity in arms, legs, and back. This value refers to the maximum value registered among all the muscles of the area to be analyzed, regarding to the maximum effort that said muscle can performing/support.

**Figure 6:**
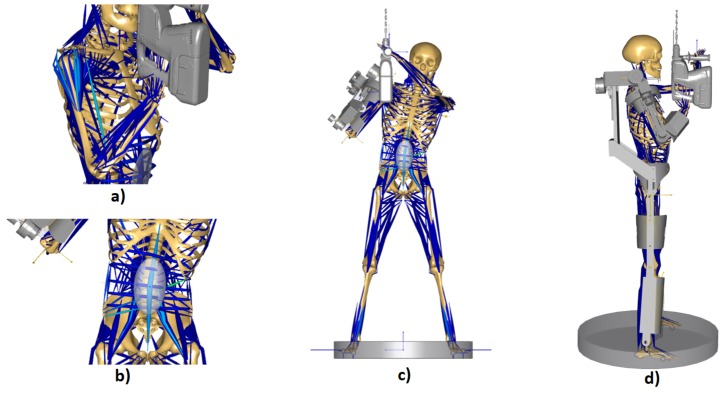
(**a**) Detail of the most loaded muscles in the simulation without the exoskeleton. (**b**) Detail of the most loaded muscles in the simulation with the upper-limb exoskeleton. (**c**) Simulation with the upper-limb exoskeleton. (**d**) Simulation with the full exoskeleton.

In the first case, the position of the user has been restricted by feigning the task of drilling an object located in the ceiling. To do this, a tool model is imported into AnyBody™ to make a more realistic simulation, and the efforts generated in the worker’s joints are analyzed. In Figure 6a, the simulation model result can be observed, and it can be seen the muscles that are subjected to the greatest effort on a scale from dark blue to green, on which the muscles in dark blue are the least loaded and in green the most.

In the second simulation, all the elements that make up the exoskeleton have been exported to .stl files, which are then imported into AnyBody™. To be able to simulate the musculoskeletal analysis, the system must be well restrained on the user, so that the results are as close to reality as possible.

Comparing the results shown in Table 3, it can be seen that the efforts supported by the worker increase considerably. Since the mechanism is not actuated, it is the user who makes all the effort to move it. Thus, to the efforts that were made previously adds the weight of the device.

Next, it proceeds to act the exoskeleton to reduce the weight that falls on the operator. To do this, it is simulated that the motors of the mechanism perform such torques that counteract the efforts previously produced in the joints. The torques that have been introduced in the simulation are the following:

- Shoulder abduction/adduction (sA/A) →−12 Nm
- Shoulder flexion/extension (sF/E) →−12 Nm
- Shoulder internal/external rotation (sI/R) → 7.5 Nm
- Elbow flexion/extension (eF/E) →−4 Nm

As can be seen in Table 3, the reduction of efforts is considerable. However, the percentage of muscle activity in the arms, legs, and back is still too high, as can be seen in Figure 6b,c. This is, as it has been described throughout the article, due to the weight that is introduced by the incorporation of the upper-limb exoskeleton. Because of this, it is proposed to add a lower-limb exoskeleton that transmits the weight of the robotic system to the floor.

We proceeded, therefore, to import the lower-limb exoskeleton model into AnyBody™ and analyze the reduction of the user’s muscle activity, as shown in Figure 6d.

Comparing the results obtained in both cases, Table 3, an improvement can be observed in terms of the reduction of the efforts endured by the worker, as well as a considerable decrease in the percentage of muscular activity in the arms, legs, and back. This implies less suffering in the muscles and joints of the operator throughout his working day, which translates into a lower probability of suffering musculoskeletal disorders in the future.

It is necessary to comment that there is an increase in the muscular activity of the arm with respect to the simulation without the exoskeleton. Since the device does not allow movements in the clavicle, additional loads appear in the muscles of this area due to limitation in the movement. To avoid this, it is proposed to add in future versions, an additional degree of freedom that allows the movements of the scapula, providing greater comfort to the user.

Even though the efforts to be made by the operator have decreased significantly, it is intended to minimize such efforts calculating the torque that should be provided by the actuators of the robotic system.

For this purpose, the joints of the device have been blocked in AnyBody™ to calculate the torque that motors should provide to guarantee a practically null effort of the user. These moments are shown below, and the efforts that appear as a result in the joints are shown in Table 3.

- Shoulder abduction/adduction (sA/A) →−12.2 Nm
- Shoulder flexion/extension (sF/E) →−16.8 Nm
- Shoulder internal/external rotation (sI/R) → 8.2 Nm
- Elbow flexion/extension (eF/E) →−7.5 Nm

Therefore, applying these torques in each of the motors, it is demonstrated that the worker hardly makes efforts to carry out the analyzed task, and also that the actuators are below their nominal value.

## 3. Upper-Limb Exoskeleton Prototype: Electromyography Analysis

In this section, an experimentation is carried out in the laboratory where different users will replicate the simulated task in the previous validation, both with the help of the exoskeleton and without it, with the objective of collecting and comparing the EMG signals produced by some key muscles during the activity. The main objective of this experimentation is to verify that there is a reduction in the muscular activity when the user executes the task with the help of the robotic device, with which we could say that the proposed system is a possible solution to reduce musculoskeletal disorders in industrial tasks.

### 3.1. Materials and Methods

#### 3.1.1. Subjects

10 healthy subjects participated in this study. The study group consists of 8 men and 2 women, all right-handed, with ages between 23 and 35 years (28.8 years ± 3.4 years), with heights ranging between 1.63 and 1.83 m (1.733 m ± 0.064 m), and a weight between 56 and 86 kg (72.32 kg ± 11.97 kg).

#### 3.1.2. Experimentation Setup

Depending on the condition, subjects sat in front of a screen wearing or not the arm exoskeleton in their dominant arm. A graphical interface was employed to give instructions to the user, as is explained in sections below.

#### 3.1.3. Acquired Data

**Figure 7:**
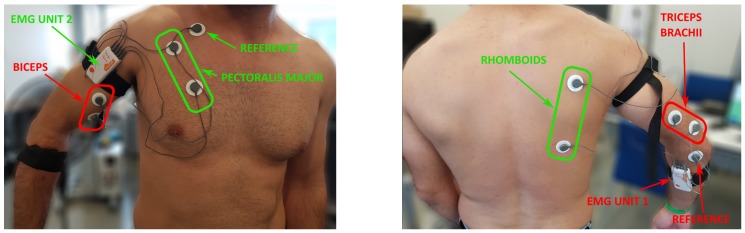
Disposition of the electrodes used to obtain the EMG signal in upper-arm and torso. *The participant that appears in the figure has provided written and informed consent for its publication.*

For this experimentation, two Shimmer3 EMG units were employed [36]. Each of these devices has 2 channels. One of the devices were used to monitor the upper-arm area (biceps and triceps brachii) and a second one for the torso area (the pectoralis and the rhomboids). The references were placed on the epitrochlea in the upper-arm and in the clavicle for the torso. Figure 7 shows the configuration of the electrodes used, where the green ones correspond to the device that records the muscle signals in the torso, and the red ones correspond to the device that collects the muscle signals in the upper-arm.

The output measure of Shimmer3 EMG unit is the level in millivolts of the Electromyography (EMG) measures muscle response, in other words, the electrical activity in response to a nerve’s stimulation of the muscle. Signal was sampled at a sampling rate of 1 kHz. Signals were processed setting an 8-degree high pass filter at 15 Hz to remove the continuous component of the EMG signal. After that, the upper envelopes of the signal were extracted.

**Figure 8:**
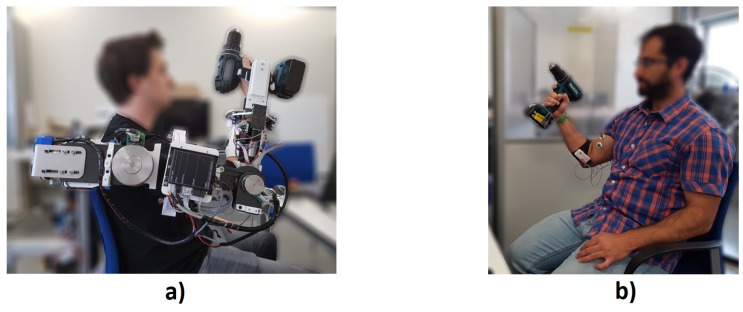
(**a**) Task without the upper-limb exoskeleton. (**b**) Task with the upper-limb exoskeleton. *All the participants that appear in the figure have provided written and informed consent for its publication.*

In addition to the Shimmer3 EMG units, the other element used in the experimentation is the prototype of the upper-limb exoskeleton shown in Figure 8, of which no data have been recorded on this occasion.

#### 3.1.4. Study Protocol

First, the subject is explained what the experimentation consists of, and the electrodes are placed for the capture of the EMG signals, as described in the previous section. Once it has been verified that the communication between the devices has been done correctly, we begin the experimentation. The task is to perform a movement of raising the arm with a drill above the head, performing a shoulder flexion of 90 degrees and maintaining at all times an angle between the arm and the forearm of 90 degrees (elbow flexion).

There are two conditions that have been carried out in a random way: the completion of the task with the help of the upper-limb exoskeleton (Figure 8a), and the execution of it without robotic device (Figure 8b).

There will be 15 repetitions in each condition, first without the tool to establish a base, and then loading a 1.7 kg drill with the right arm. Each of these repetitions is divided into four phases:

- Warning message (1 s)
- Raise the arm (5 s)
- Maintenance of the arm in vertical working position, with a shoulder and elbow flexion of 90 degrees (3 s)
- Lowering the arm (5 s)

**Figure 9:**
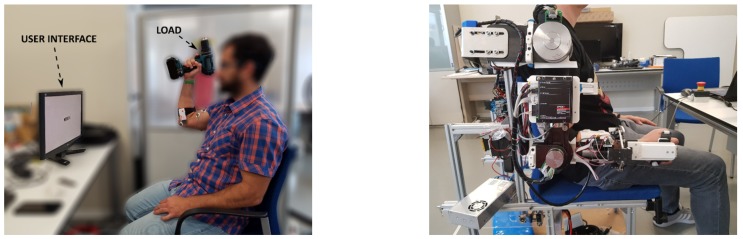
Experimentation setup. *All the participants that appear in the figure have provided written and informed consent for its publication.*

To control these times when the task is executed without the exoskeleton, the subject has an interface that tells him when to start and stop each of the movements (Figure 9), which allows us to have indicated each of the events performed on the EMG signal obtained.

The subjects performs the experimentation in a sitting position, and the weight of the exoskeleton is supported by a structure of aluminum commercial profiles fixed to the ground, since we do not have the prototype of the passive lower-limb exoskeleton to transmit said weight to the ground.

**Figure 10:**
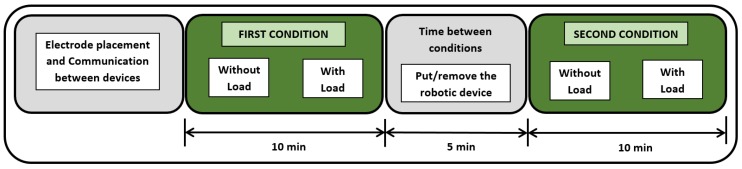
Study protocol.

The average duration of the experimentation is about 40 minutes per subject, since the task to be performed is explained until the last repetition ends and the electrodes are removed. Figure 10 summarizes the steps taken to perform the experimentation.

#### 3.1.5. Statistical Analysis

The average value of the EMG signal has been extracted for each of the muscles of every subject. This value has been obtained by means of the 15 average values of the EMG signal corresponding to the 15 repetitions of each condition.

For the study of the differences between the four conditions for each of the 4 muscles, one-way repeated-measure ANOVA was employed. Holm adjustment was applied in the pairwise comparison between conditions. Kolmogorov-Smirnov and the Shapiro–Wilk tests, were considered.

### 3.2. Results

#### 3.2.1. Biceps

**Figure 11:**
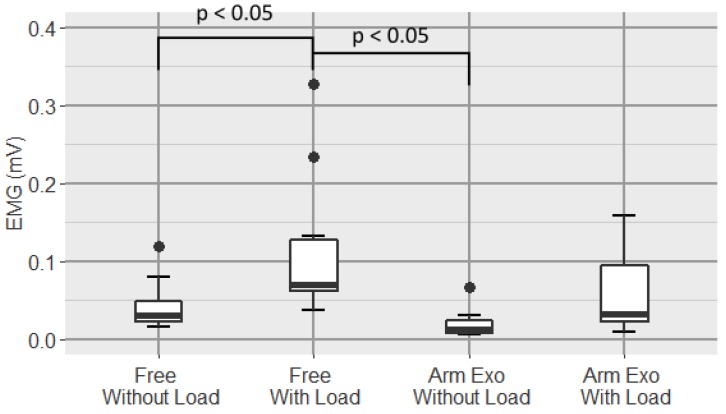
Boxplot of the EMG signal of the biceps for all conditions.

A boxplot of the average of the EMG signal of the biceps is shown in Figure 11.

Analysis shows a very high significant differences between conditions (one-way repeated-measure ANOVA p=0.0005). In the pairwise comparisons we found a significant difference between both free conditions (p=0.0327) and a large statistical difference between the Free With Load and Arm Exoskeleton Without Load conditions (p=0.0031).

#### 3.2.2. Triceps Brachii

**Figure 12:**
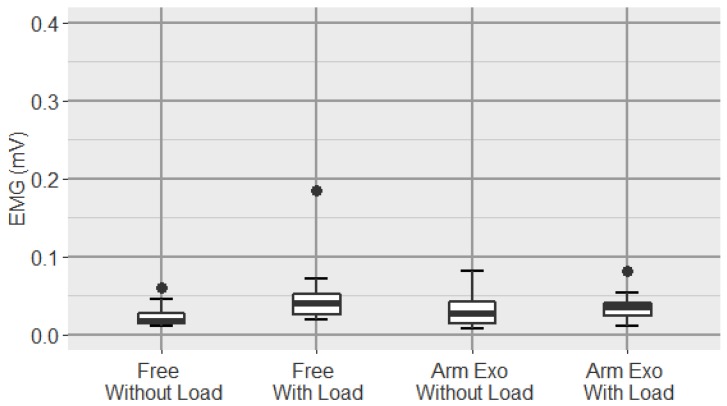
Boxplot of the EMG signal of the triceps brachii for all conditions.

Regarding triceps brachii (Figure 12), although the ANOVA test is positive (one-way repeated-measure ANOVA p=0.046), in the pairwise comparision, we did not find significant differences between the different conditions.

#### 3.2.3. Pectoralis

**Figure 13:**
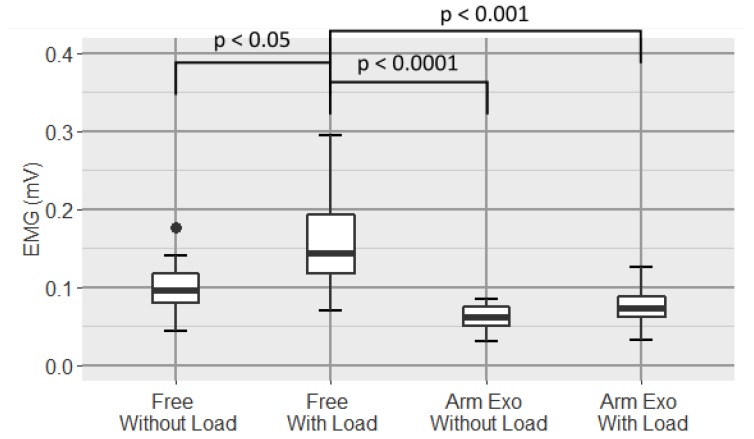
Boxplot of the EMG signal of the pectoralis major for all conditions.

In the case of pectoralis (Figure 13), one-way repeated-measure ANOVA shows a large statistical difference ($p=9.89\times 10^{-8}$). There is a significant difference between both free modes (p=0.015), and there is also a high significant difference for the Free With Load and Arm Exoskeleton Without Load conditions pair ($p=4.5\times 10^{-5}$) and also between Free With Load and Arm Exoskeleton With Load conditions (p=0.0004).

#### 3.2.4. Rhomboids

**Figure 14:**
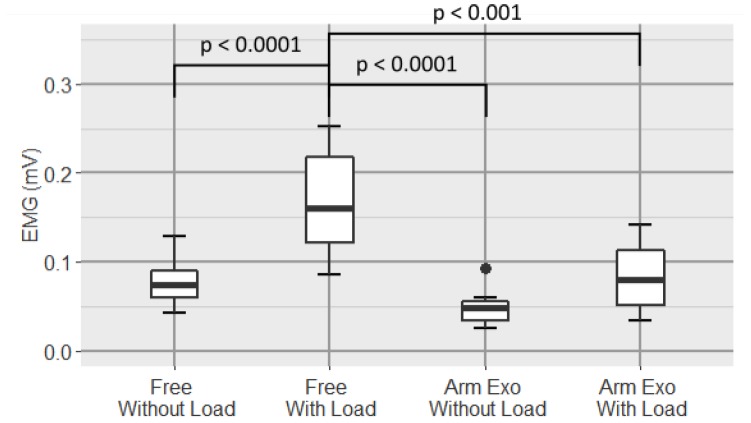
Boxplot of the EMG signal of the rhomboids for all conditions.

Figure 14 shows a boxplot of the average of the EMG signal of the rhomboids.

In this case, one-way repeated-measure ANOVA shows a very high significant difference between groups ($p=1.48\times 10^{-10}$). In the pairwise comparison we found very high significant differences between both free conditions ($p=6.2\times 10^{-5}$), Free With Load and Arm Exoskeleton Without Load conditions pair ($p=4.5\times 10^{-7}$) and Free With Load and Arm Exoskeleton With Load conditions pair (p=0.00012).

### 3.3. Discussion

In Figure 11 it can be seen that there is a statistically significant difference between the EMG levels of the biceps between both free conditions, which implies that the difference in biceps muscle effort between carrying weight and not is significantly different. However, between carrying weight and not while wearing the arm exoskeleton, although a difference is observed, this is not significant. In addition, as it can be seen in the figure, levels are lower, respectively, in the cases in which the exoskeleton is worn, which indicates that the active upper-limb exoskeleton is effective to reduce the muscle activity in the biceps. Biceps is the only of four measured muscles which acts as a tonic muscle, the triceps branchii, the pectoralis and the rhomboids acts as phasic muscles.

On the other hand, in the case of the EMG levels of the triceps brachii muscle (Figure 12), no difference between conditions was observed. From the data obtained, it can be deduced that the activity observed in this muscle is mainly due to the grasp of the load with the hand. Because of this, no reduction is observed when using the exoskeleton.

With respect to the pectoralis and the rhomboids, a very high reduction in activity of both muscles in the cases of using the exoskeleton can be observed. First, as with the biceps, it can be seen that for the pectoralis and the rhomboids there are also a statistically significant difference between both free modes but there is not a statistically significant difference between carrying weight and not while wearing the upper-limb exoskeleton. However, unlike the biceps, it is observed that the increase in muscle activity when carrying weight with the exoskeleton compared to not carrying weight with exoskeleton is much lower in these muscles (pectoralis and rhomboids). In addition, it can be seen that the reduction caused in the case of carrying the load with the exoskeleton with respect to carrying the load without wearing it, is very significant, which is a clear indicator that the use of the proposed active upper-limb exoskeleton is very effective for the reduction of muscle activity in pectoralis and rhomboids muscles.

Based on the data, not only is reduced the average muscular level measured by the amplitude of EMG signals, but also the variability is considerably reduced, reaching values close to half the ones obtained for the condition without exoskeleton (from 0.159±0.067 to 0.077±0.027 in the case of the pectoralis and from 0.167±0.060 mV to 0.083±0.0382 mV in the case of the rhomboids).

## 4. Conclusions

This paper aims to validate the proposed solution to the problem of musculoskeletal disorders existing in industry due to overstress and tiring postures taken by operators in their jobs.

First, the design of the robotic system has been validated in a simulation environment for subsequent manufacturing. After analyzing the data we extracted from the simulation carried out with AnyBody™ software, we can conclude that the efforts supported by the operators in certain industrial tasks decrease wearing the proposed upper-limb exoskeleton.

Once confirmed that the designed device supposes a benefit for the user, we proceed to the manufacture and validation of the prototype of the upper-limb exoskeleton based on the previous analyzed design. To do this, a laboratory experiment was carried out in which the EMG signals collected when performing an industrial activity with and without the help of the robotic system are compared. After analyzing the results obtained in such experimentation, we can conclude that the prototype of the proposed active upper-limb exoskeleton reduces muscle activity and therefore the efforts supported by the user during the performed task determined by the amplitude of the recorded EMG signals.

MovilFrio is satisfied with the results obtained and discussed in this paper, since it considers that the incorporation into the company of the developed robotic exoskeleton could be beneficial for its workers. The company has shown its interest in the development of the passive lower-limb structure that would support the weight of the upper-limb exoskeleton, since it would mean a greater reduction in musculoskeletal disorders suffered by the operators, and also, it proposes us to investigate what would happen if we added an end-effector to the robotic device that would allow manipulation of tools of a larger size and/or weight.

## 5. Ethics Statement

The Institutional Review board at Miguel Hernandez University approved the experimental validation of ExIF project (2016.06.16.FPRL). All participants provided written and informed consent before their participation in the experiments.

## Figures and Tables

**Table 1:** Maximum torque and power, including the estimated weight of the exoskeleton.

Movement	Maximum Torque (Nm)	Maximum Power (W)
Shoulder abduction/adduction (sA/A)	21	18
Shoulder flexion/extension (sF/E)	42	63
Shoulder internal/external rotation (sI/E)	18	27
Elbow flexion/extension (eF/E)	30	33

**Table 2:** Joint limits of the robotic system.

Angle	Lower Limit	Upper Limit
$q_{0}$	−9°	160°
$q_{1}$	−43° + $q_{0}/3$	153°−$q_{0}/6$
$q_{2}$	−60°—$q_{0}\cdot 4/9$ + $q_{1}\cdot 5/9-q_{0}q_{1}\cdot 5/810$	90°—$q_{0}\cdot 7/9$ + $q_{1}/9-q_{0}q_{1}\cdot 2/810$
$q_{3}$	−90°	60°
$q_{4}$	−90°	90°

**Table 3:** Results obtained from the inverse dynamic analysis in AnyBody™ for the musculoskeletal model of the human body.

	Operator without Exoskeleton	Non-Assisted Arm Exoskeleton	Assisted Arm Exoskeleton	Active Full Exoskeleton	Joint Block Arm Exoskeleton
[c]Shoulder
abduction/adduction
(sA/A) [Nm]	2.27	9.97	1.26	1.20	0.089
[c]Shoulder
flexion/extension
(sF/E) [Nm]	12.83	10.23	4.93	4.63	−0.082
[c]Shoulder
internal/external rotation
(sI/E) [Nm]	6.12	7.58	0.12	0.01	−0.04
[c]Elbow
flexion/extension
(eF/E) [Nm]	3.32	4.03	2.66	2.69	−0.04
[c]Max. Arm
Muscle Activity [%]	3.90	42.20	20.67	14.00	17.27
[c]Max. Legs
Muscle Activity [%]	5.33	6.57	22.82	1.78	2.00
[c]Max. Torso
Muscle Activity [%]	18.61	23.00	44.00	2.37	2.77
